# Real‐world data of a digitally enabled, time‐restricted eating weight management program in public sector workers living with overweight and obesity in the United Kingdom: A service evaluation of the Roczen program

**DOI:** 10.1002/osp4.730

**Published:** 2024-02-09

**Authors:** Adrian Brown, Laurence J. Dobbie, Laura Falvey, Dipesh C. Patel, Jonathan T. C. Kwan, Siri Steinmo, Ling Chow, Barbara M. McGowan

**Affiliations:** ^1^ Centre for Obesity Research University College London London UK; ^2^ National Institute of Health Research London UK; ^3^ Reset Health Ltd London UK; ^4^ Diabetes and Endocrinology Department Guys Hospital Guys & St Thomas's Hospital London UK; ^5^ Division of Medicine University College London London UK; ^6^ Diabetes and Endocrinology Department Royal Free NHS Trust London UK; ^7^ Darent Valley Hospital Dartford Kent UK; ^8^ Critical Care Unit University College London NHS Hospital London UK

**Keywords:** digital‐health, obesity, time‐restricted eating, work based health

## Abstract

**Introduction:**

The health of the United Kingdom workforce is key; approximately 186 million days are lost to sickness each year. Obesity and type 2 diabetes (T2D) remain major global health challenges. The aim of this retrospective service evaluation was to assess the impact of a digitally enabled, time‐restricted eating (TRE) intervention (Roczen Program, Reset Health Ltd) on weight and other health‐related outcomes.

**Methods:**

This service evaluation was conducted in people living with overweight/obesity, with 89% referred from public sector employers. Participants were placed on a TRE, low‐carbohydrate, moderate protein plan delivered by clinicians and mentors with regular follow up, dietary guidance, goal setting, feedback, and social support.

**Results:**

A total of 660 members enrolled and retention was 41% at 12 months. The majority were female (73.2%), 58.9% were of White ethnicity, with a mean (SD) age of 47.5 years (10.1), and a body mass index of 35.0 kg/m^2^ (5.7). Data were available for 82 members at 12‐month. At 12‐month, members mean actual and percentage weight loss was −9.0 kg (7.0; *p* < 0.001) and −9.2% (6.7, *p* < 0.001) respectively and waist circumference reduced by −10.3 cm (10.7 *p* < 0.001), with 45.1% of members achieving ≥10% weight loss. Glycated hemoglobin was significantly improved at 6 months in people living with T2D (−11 mmol/mol [5.7] *p* = 0.012). Binge eating score significantly reduced (−4.4 [7.0] *p* = 0.006), despite cognitive restraint increasing (0.37 [0.6] *p* = 0.006).

**Conclusion:**

Our service evaluation showed that the Roczen program led to clinically meaningful improvements in body weight, health‐related outcomes and eating behaviors that were sustained at 12‐month.

## Graphical abstract

This service evaluation showed the efficacy of the Roczen program in public sector workers living with overweight and obesity.

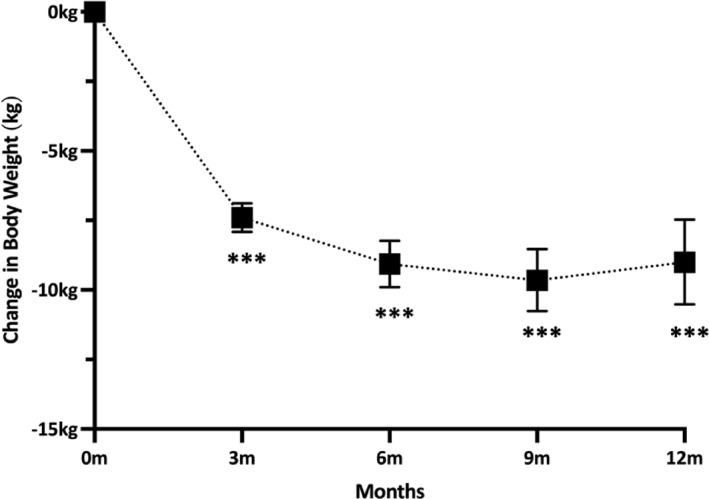

## 1 INTRODUCTION

Workplace health is essential, especially within public services such as healthcare and transport, where staffing levels affect service delivery and public safety. Unemployment is associated with an increased risk of cardiovascular disease, poorer mental health, and early death.^1^ The estimated cost of sickness absence and worklessness in the United Kingdom (UK) is over £100 billion per year, which is greater than the annual cost of running the National Health Service (NHS).^2^ Data shows that in the UK, in 2022, 185.6 million workdays were lost to sickness absence, with a record high of 104.9 million of these days lost to those living with long term conditions.^3^ People living with obesity have been shown to have an additional four extra absence days per year.^4^ NHS workforce data showed sickness absence was over 2.4 million workdays in March 2022.^5^ Staff absences are higher amongst clinically qualified staff, including ambulance workers, doctors and nurses, with mental health, musculoskeletal issues and cardiovascular disease being reported as key reasons.^5^ Well‐being and stress also predict sick leave and productivity level.^6^ Furthermore, obesity is linked to 2–3 times higher levels of extended work absence^7^ and is associated with mental health and musculoskeletal conditions.^8^ This has resulted in both the government and employers introducing workplace health interventions to improve the health of the workforce. Despite this, there remain challenges in maintaining workplace health.

Obesity and type 2 diabetes (T2D) are significant global public health concerns,^9^ with data showing that 44% of public sector workers are living with overweight or obesity.^10^ Interventions proven to help manage both conditions include lifestyle intervention, pharmacotherapy and bariatric and metabolic surgery.^11^ However, access to services for treatment in the UK remains limited, with only 60% of regions in England having access to Specialist Obesity services (Tier 3),^12^ resulting in potential health inequalities based on postcode.^13^

Digital health tools are increasingly used to assist patients in chronic disease self‐management, and form an integral part of the NHS long‐term plan.^14^ These have been shown to improve health behaviors, increase compliance with treatment^15^ and may improve access to healthcare.^16^ These interventions may also be applicable to address workers' health, with digitally delivered occupational health interventions shown to improve worker well‐being and reduce metabolic risks.^17^, ^18^ Therefore, digital health interventions may offer a novel solution to improve healthcare access and improve the health of the public sector workers.

Continuous energy restriction can help people lose weight; however, there is significant variability in response and more recently time‐restricted eating (TRE) has increased in popularity.^19^ TRE is a form of intermittent fasting involving a period of fasting and eating within each 24‐h period for example, 16:8 (16 h fasting and 8 h eating).^20^ Substantial evidence exists supporting the effectiveness of TRE in achieving substantial weight loss, cardiometabolic health, and mental health.^21^, ^22^ Despite this, there remains limited evidence evaluating a digital TRE program in patients living with overweight and obesity, particularly in the real world and in a workplace setting. This has considerable potential to lead to improvements in health and adherence to intervention and specialist healthcare access whilst offering a person‐centered and personalized approach. Therefore, a retrospective service evaluation was conducted of the impact of the Roczen program on body weight (BW), surrogate markers of cardiovascular disease, and other‐health related indices in people living with overweight and obesity.

## 2 METHODS

A retrospective service evaluation was conducted to assess the impact of a digitally enabled TRE intervention (Roczen Program, Reset Health Ltd) on people living with overweight/obesity, the majority of whom enrolled, via self‐referral, to an employer‐led health incentive program. For those who enrolled via their employer, their place on the intervention was paid for (by the employer) for 12 months. Shift‐workers were allowed to enroll. These individuals, upon confirmation of eligibility and onboarding, became members of the Roczen program.

### 2.1 Roczen program

The Roczen Program is a medically‐led, technology‐enabled weight management program (https://www.roczen.com/). It has been designed by a multidisciplinary team of specialists to help people living with obesity and/or T2D and other complications to improve metabolic health, quality of life, and reduce associated disease risk. Members can use the Roczen Mobile App to message the clinical team at any time and are followed up every 4‐week by a clinician via telephone or video consultation. The program is unique as it offers mentoring for peer‐led social support via the App and regular feedback is provided to the members through varied modalities of clinical interactions.

The program utilizes TRE alongside low‐carbohydrate and moderate protein intake (see Supporting Information S1 for more detail on mentor scheme and program development). Energy intake monitoring and measurement of macronutrient composition are not specified to members. The program incorporates additional evidence‐based behavior change techniques (BCTs), including goal setting paired with self‐monitoring, motivational interviewing, habit formation, incentivized mentor scheme, and educational resources.^23^ These focus on engaging and empowering the patient in decisions that impact their health whilst highlighting the health consequences of chronic disease, practical behavioral instruction on realistic lifestyle changes, and the benefits of optimal obesity and diabetes management.^24^, ^25^ Over the first 12 weeks, members were recommended to increase physical activity (PA) from their baseline and reduce sedentary behavior. All members were provided with videos detailing foundational movements. Ongoing PA was supported throughout the program with goal‐setting and self‐monitoring to add specific exercises or activities that the patient found acceptable and beneficial. There was no specification on the type of PA prescribed as various forms of PA can benefit health.^26^ Prior to commencing the Roczen program, participants were assessed Binge Eating Disorder (BED) to ensure safety using the Binge Eating Scale (BES) and, if required, clinical interview.^27^

### 2.2 Member pathway

The member pathway is initiated with a self‐referral and sign‐up process where members who are eligible at this stage give consent for the medical team to access their NHS Summary Care Records. The individual then gains access to the Roczen Mobile App, and the clinician is alerted to the prospective member in the web‐based Roczen Clinic App.

The medical team arranges a video consultation and sends digital versions of validated health questionnaires to assess mental health, eating behaviors, the risk of sleep apnea and binge eating. If they remain eligible, members are onboarded in their first video consultation and assigned a peer‐to‐peer mentor. Together, the clinician and member agree on an initial 12‐week plan. The timing of eating and fasting was tailored to the individual and based on patient preference, but members were encouraged to retain the same TRE pattern where feasible. They then received written materials with guidance on TRE, nutrition, and BCTs. Additionally, they are given personalized content for other relevant conditions or considerations which may impact progress, such as shift work or menopause.

Members were not advised to eat ad libitum, and instead were advised to eat two discrete meals with a break in between where snacking was discouraged. The most common TRE pattern was a fasting window between 8 and 12 PM the next day, constituting a 16 h fast, with the first meal between 12 and 2 PM and the second meal between 6 and 8 PM. Regardless of personal preference, all members were encouraged to finish eating before 8 PM.

All new members were facilitated to undergo an HbA1c blood test for the purpose of T2D screening, via a postal capillary blood testing kit.

Members have ongoing biometrics monitoring and follow‐up facilitated via the Roczen app, through both asynchronous messaging and synchronous video consultations with clinicians.^28^ Adherence was assessed by clinicians during virtual clinical consultations. Some members volunteer to become mentors via an educational program provided by Roczen, others continue on the program to aid maintenance, and others leave by choice. Within the program, members were not advised to leave or be discharged by the clinical team at any set point, in keeping with the chronicity of obesity and T2D. For members with T2D treated with insulin, mentoring was provided by a clinician and monitoring requirements stipulated were more frequent, including weekly weights for the first 12 weeks rather than monthly as per the weight management pathway. Mentors were encouraged to continue their own program whilst providing dedicated peer‐to‐peer support to other members on the program, and were incentivized through a point‐based reward system.

### 2.3 Outcome measurement

The primary outcome was BW change at 12 months from baseline. The percentage of participants who achieved less than 5%, more than 5%, >10, >15 and >20% of weight loss were categorized. Secondary outcomes were anthropometry including waist circumference (WC), surrogate markers of cardiometabolic health including glycated hemoglobin (HbA1c), systolic blood pressure (SBP), diastolic blood pressure (DBP), and mental health and eating behavior. Members' mental health and eating behavior were collected using the following validated questionnaires: Patient Health Questionnaire (PHQ‐9), Generalized Anxiety Disorder (GAD‐7), Three Factor Eating Questionnaire (TFEQ), and BES.^29^, ^30^, ^31^, ^32^

### 2.4 Eligibility criteria for the program

To be eligible for the program, individuals had to be ≥18 years old with minimum body mass index (BMI) criteria defining overweight related to ethnicity specific cut‐offs.^11^ Further screening was carried out to ensure members with exclusionary conditions such as type 1 diabetes, pregnancy, eating disorder diagnosis and suicidal ideation were not included for safety and increased risk (further details are supplied in Supporting Information S1).

### 2.5 Data collection

Data were self‐reported to clinicians during review appointments or via the Roczen app. Members were advised on how to accurately measure key outcomes such as weight, WC, or BP. Due to the COVID‐19 pandemic, there were challenges obtaining anthropometric, BP, and other outcome data during this period. Baseline measurements for BW, BMI, WC, and BP were deemed acceptable ±2 weeks of plan initiation. An HbA1c measurement was considered acceptable between −12 weeks and +4 weeks following plan initiation. This timeframe was agreed on a pragmatic basis given the real‐world nature of the service and its evaluation. Furthermore, HbA1c measurement was assessed in addition to a clinical consultation, noting possible symptoms or signs suggesting uncontrolled diabetes, which would prompt delayed onboarding or reassessment.

**FIGURE 1:**
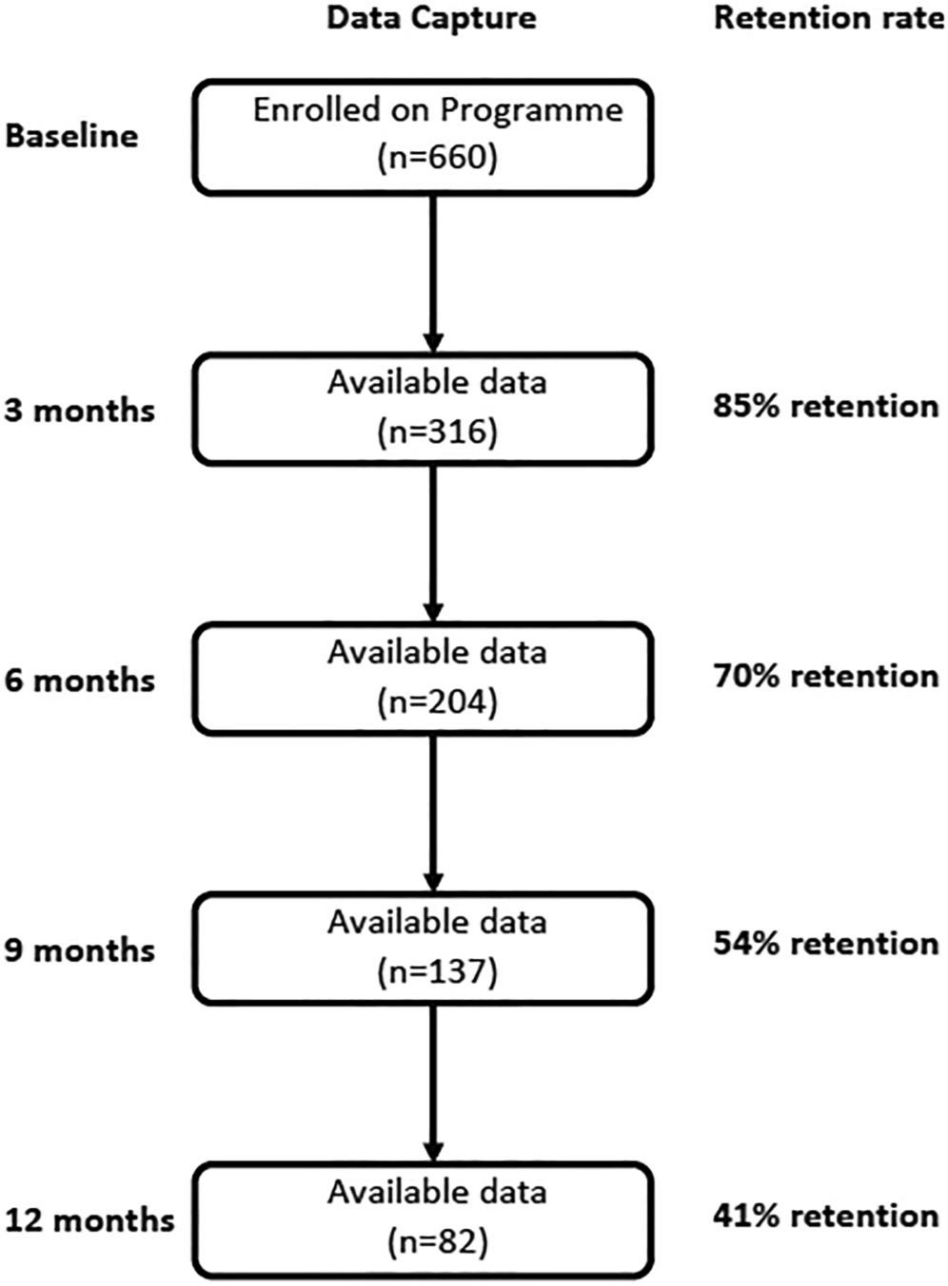
Members data capture and retention rate at each time point over 12‐month. *n*, number; %, percentage.

Data were available for 82 members who submitted their data at 12 months and will be described as 12‐month respondents within the analysis this is not reflective of the retention rate but rather the available data at this time point for analysis. HbA1c outcomes are reported at 3 and 6 months only for people living with T2D based on the availability of data at the time of analysis. Note that as this was a rolling program, the available data at each time point does not signify the retention rate of the program but rather indicative of those members who had successfully submitted data within the acceptable time frames (Figure 1).

### 2.6 Statistics analysis

Statistical analysis was performed on SPSS Statistics (IBM, Version 26.0) with all graphs being created using GraphPad Prism. Statistical significance was defined as a *p*‐value <0·05. As this was a service evaluation, no formal sample size calculation was conducted. Demographic data were summarized using mean (standard [SD]) for parametric data and median (interquartile [IQR]) for non‐parametric data. Continuous data with categorical data were summarized using counts (percentages). To assess the differences in pre‐ and post‐intervention, parametric and non‐parametric data were analyzed using appropriate tests (*t*‐test; paired *t*‐tests; Mann Whitney‐*U* test; Friedman) and one way Analysis of Variance was performed to see if T2D and prediabetes, sex, or living with obesity impacted on weight loss outcome. Appropriate log transformation was performed. Bonferroni adjustment was used for multiple comparisons.^33^ Binomial logistic regression was used to assess predictors of members completing the program with sex, ethnicity, age, baseline weight, PHQ‐9 and TFEQ sub‐scales were used as control variables. Odd ratios, 95% confidence intervals and *p*‐values were reported for significant outcomes. The assumptions of each model were checked and met.

### 2.7 Ethics

This was a retrospective service evaluation reviewing previously collected data from the Roczen member and therefore required no formal ethical approval.

## 3 RESULTS

In total, 660 were eligible and enrolled with the Roczen program (Figure 1, Table 1). The reasons for members not enrolling were due to medically ineligible, individuals changed their mind, or not respond to initial invitation correspondence. Overall retention to the Roczen program was 41% at 12 months, with retention at 3, 6, and 9 months being 85%, 70% and 54%, respectively (Figure 1).

**TABLE 1:** Baseline characteristics for enrolled Roczen members and 12‐month respondents.

Characteristics, *n*	Enrolled members (*n* = 660)	12 m respondents (*n* = 82)
Men, *n* (%)	177 (26.8)	17 (20.7)
Women, *n* (%)	483 (73.2)	65 (79.3)
Age, years (SD) (*n* = 600)	47.5 (10.1)	50.0 (9.4)
Ethnicity, *n* (%) (*n* = 645)		
Black African	70 (10.9)	8 (10.1)
Black Caribbean	43 (6.7)	5 (6.3)
Chinese	4 (0.6)	1 (1.3)
Indian	72 (11.2)	13 (16.5)
Middle Eastern	8 (1.2)	1 (1.3)
Mixed	18 (2.8)	3 (3.8)
Other	31 (4.8)	5 (6.3)
Pakistani	10 (1.5)	1 (1.3)
White	389 (60.3)	42 (53.2)
Bodyweight, kg (SD)	97.4 (19.0)	98.1 (20.3)
BMI, kg/m^2^ (SD)	35.0 (5.7)	35.8 (6.5)
Waist circumference, cm (SD)	107.0 (14.0)	108.1 (14.5)
Public sector workers, *n* (%)	585 (88.6)	73 (89)
Metabolic classification, *n* (%) (*n* = 641)		
Overweight	60 (9.1)	7 (8.5)
Obesity	442 (69.0)	53 (64.6)
Prediabetes	63 (9.8)	12 (14.6)
T2D	76 (11.9)	10 (12.2)
Living with obesity, *n* (%) (*n* = 595)	487 (81.8)	66 (81.5)
HbA1c, mmol/mol (*n* = 467)	39.9 (8.3)	39.8 (7.0)
Systolic blood pressure, mm Hg (SD)	127.2 (13.5)	128.7 (15.7)
Diastolic blood pressure, mm Hg (SD)	79.6 (9.6)	80.9 (11.2)
Hypertension, *n* (%) (*n* = 373)	31 (8.3)	8 (13.3)
Mental health and eating behavior scores		
PHQ‐9 (*n* = 550)	4.0 (2.0, 8.0)	3.5 (1.0, 6.0)
GAD‐7 (*n* = 550)	3.0 (1.0, 7.0)	3.0 (1.0, 6.0)
BES (*n* = 458)	12.9 (8.0)	13.0 (6.5, 17.5)
TFEQ total (*n* = 546)	45.0 (13.6)	44.6 (11.5)
TFEQ restraint	2.23 (0.6)	2.32 (0.6)

*Note*: Data are in *n* (%), mean (SD).

Abbreviations: %, percentage; BES, Binge Eating Scale; BMI, body mass index; cm, centimeter; GAD7, Generalized Anxiety Disorder Questionnaire 7; HbA1c, glycated hemoglobin; IQR, interquartile range; kg, kilograms; kg/m^2^, kilograms per meter squared; m, months; Median (IQR) *n*, number; mmol/L, millimoles per liter; mm Hg, millimeter of mercury; PHQ‐9, Patient Health Questionnaire 9; SD, standard deviation; T2D, type 2 diabetes mellitus; TFEQ, three factor eating questionnaire.

Of those enrolled, the mean age was 47.5 years (SD 10.1), 73.2% (*n* = 483) were females, 58.9% were of White ethnicity (*n* = 389), 81.8% were living with obesity (*n* = 487), 9.8% with prediabetes (*n* = 63), and 11.9% with T2D (*n* = 76) (Table 1). In terms of other measurements at baseline, BW was 97.4 kg (SD 19.0), BMI was 35.0 kg/m^2^ (SD 5.7), WC was 107.01 cm (SD 14.0), SBP was 127.2 mmHg (SD 13.5), DBP was 79.6 mmHg (SD 9.6), and median HbA1c was 38.2 mmol/mol (IQR 34.4, 43.0) (Table 1). A total of 17.1% had moderate to severe depression as per PHQ‐9 and 10.5% were with moderate to severe anxiety as per GAD‐7. From the enrolled members, 89% of members were public sector workers enrolled through staff‐wellbeing initiatives. Because of incomplete data, comparison between those registering interest for the program and enrolling was not possible.

**FIGURE 2:**
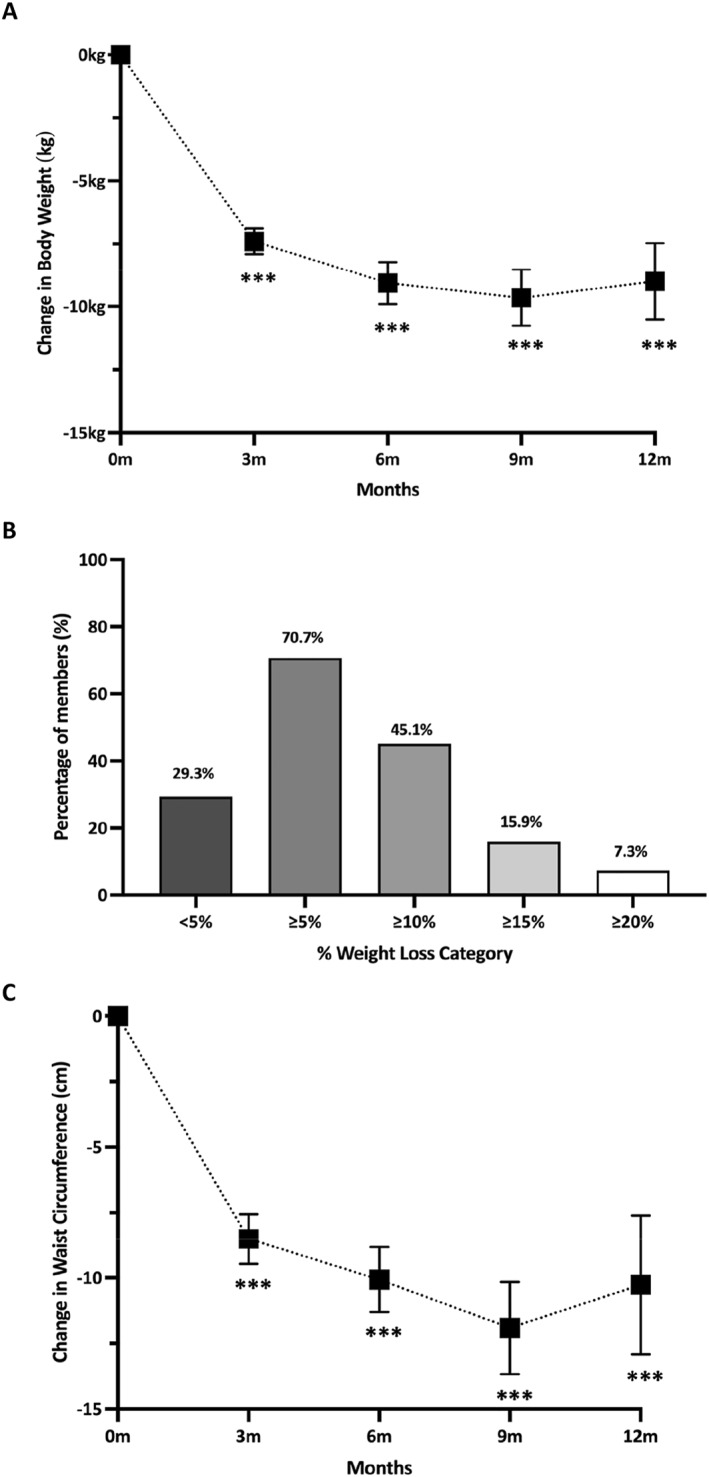
(A) Bodyweight change of members over 12 months (error bars represent 95% CIs). (B) Proportion of participants achieving categorical weight loss percentages at 12 months. kg, kilograms, %, percentage. (C) Waist circumference change of member over 12 months. m, months; kg, kilograms; and %, percentage. ***<0.001.

Outcome data were available for 82 members at 12 months. Compared to the baseline, members significantly reduced BW and percentage BW by 9.0 kg (SD 7.0, *p* < 0.001) and 9.2% (6.7, *p* < 0.001) respectively, BMI by 3.3 kg/m^2^ (SD 2.6; *p* < 0.001), and WC by 10.3 cm (SD 10.7; *p* < 0.001) equivalent to a 9.5% reduction (Table 2, Figure 2). Members also significantly reduced BW, BMI, and WC at 3, 6 and 9 months (Table S1). Further exploratory analysis assessed the impact of pre‐diabetes and T2D, living with obesity and sex on percentage weight loss at 12 months, showing that neither living with obesity, prediabetes, nor T2D impacted on percentage weight loss (Table S2). Those living with obesity had greater percentage weight loss at 3 and 6 months compared with those living with overweight but not at 9 months (Table S3). Sex impacted percentage weight loss at 12‐month with females losing more weight than males. However, no difference was seen at 3, 6 or 9 months (Table S4).

**TABLE 2:** Summary of results of available data for 82 participants: Key outcomes at 12 months.

Outcome	*n*	Baseline	12 months	Change	95% CI	*p* Value
Weight (kg)	82	98.1 (20.3)	89.1 (20.0)	−9.0 (6.9)	−10.5 to −7.5	<0.001
BMI (kg/m^2^)	82	35.8 (6.4)	32.5 (6.5)	−3.3 (2.6)	−3.9 to −2.7	<0.001
Waist circumference (cm)	65	108.1 (14.3)	97.9 (14.0)	−10.3 (10.7)	−12.9 to −7.6	<0.001
Systolic BP (mm Hg)	40	128.5 (16.6)	125.6 (13.1)	−3.0 (14.1)	−7.5 to 1.5	0.061
Diastolic BP (mm Hg)	40	81.5 (11.7)	78.5 (9.5)	−3.0 (9.7)	−6.0 to 0.1	0.185
PHQ9	28	3.5 (1.3, 5.8)	3.5 (1.3, 5.8)	−0.89	‐	0.372
GAD7	28	3.5 (0.0, 5.8)	3.5 (0.0, 5.8)	−0.40	‐	0.695
BES	28	6.8 (6.1)	7.0 (6.6)	−4.4 (7.0)	−7.5 to −1.5	0.006
TFEQ total	27	38.0 (12.1)	34.8 (13.5)	−3.2 (12.5)	−8.2 to 1.7	0.192
TFEQ restraint	28	2.20 (0.6)	2.57 (0.5)	0.37 (0.6)	0.1 to 3.0	0.006

*Note*: Data are in mean (SD).

Abbreviations: BES, Binge Eating Scale; BMI, body mass index; BP, blood pressure; CI, confidence intervals; cm, centimeter; GAD‐7, Generalized Anxiety Disorder Questionnaire 7; kg, kilograms; kg/m^2^, kilograms per meter squared; Median (IQR) *n*, number; mmHg, millimeter of mercury; mmol/L, millimoles per liter; PHQ‐9, Patient Health Questionnaire 9; TFEQ, Three Factor Eating Questionnaire.

**FIGURE 3:**
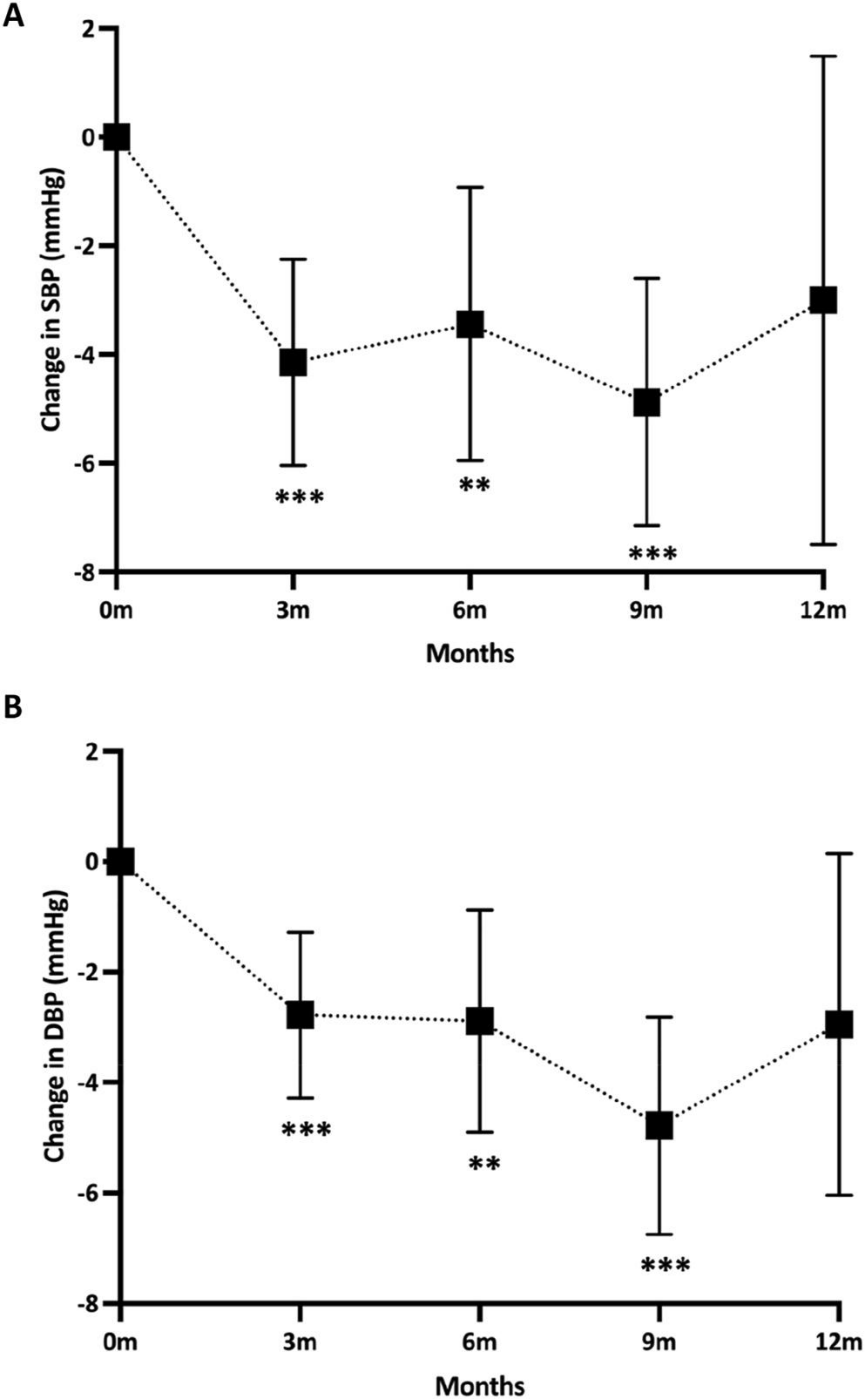
(A) Systolic blood pressure change of member over 12 months (error bars represent 95% CIs). (B) Diastolic blood pressure change of member over 12 months (error bars represent 95% CIs). m, months; mm Hg, millimeters of mercury; **<0.01; ***<0.001.

In terms of BW change at 52 weeks, 70.7% lost ≥5% BW, 45.1% lost ≥10% BW, 15.9% lost ≥15% BW, 7.3% lost 20% of BW and 29.3% lost <5% BW (Figure 3, Table 2). When missing data was imputed, weight loss remained clinically and statistically significant, albeit smaller (Table S5).

At 12‐month, SBP and DBP did not significantly reduce (−3.0 mmHg [SD: 14.1, *p* = 0.185]; −2.9 mmHg [SD 9.7, *p* = 0.06] respectively), however there were significant reductions at 3, 6 and 9 months (Table 2, Table S1, respectively). In people living with T2D, glycemic control was inadequately controlled at baseline with a mean an HbA1c of 58.8 mmol/mol (SD 6.2 IFCC) or 7.5% (SD 1.1, DCCT). At 3 and 6 months of the Roczen program, Hba1c reduced to 48.4 mmol/mol (SD 5.7) or 6.6% (SD 1.1) and 47.8 mmol/mol (SD 2.4) or 6.5% (SD 1.1), respectively (Table S6). This is equivalent to a reduction of 10.4 mmol/mol or 0.9% (*p* < 0.001) at 3 months and 11.0 mmol/mol or 1% at 6 months (*p* = 0.012).

At 12‐month, depression and anxiety scores showed no significant change (Table 2); however, differences were found at other time points over the 12‐month. At 3‐ and 6‐month depression score was significantly lower (*z* = −9.3, *p* < 0.001; *z* = −5.5, *p* < 0.001, respectively, Table S1) but there was no change at 9 months. Anxiety was significantly lower at all other time points relative to baseline (3 months *z* = −8.3; *p* < 0.001, 6 months *z* = −4.9 *p* < 0.001; 9 months −2.6, *p* = 0.009, respectively, Table S1). Over the 12‐month program, when compared to baseline, there were trends toward a higher proportion of members living with no depression (PHQ‐9) or no anxiety (GAD‐7), as well as a lower proportion of members with moderate to severe depression (PHQ‐9) (Table S7).

When looking at eating behavior, binge eating score significantly reduced between baseline and 12 months (−4.4, SD 7.0, *p* = 0.006), as well as other time points relative to baseline (Table 2), while total TFEQ eating behavior score showed no change at 12 months but significantly improved at 3, 6 and 9 months (−10.2, SD 13.8; −10.8, SD 15.4; −7.8, SD 13.2, all <0.001, respectively, Table S1). The restraint subscale of the TFEQ score was higher at 12 months (3.7, SD 6.4, *p* < 0.001), 3 and 6 months, but not at 9 months (Table S1).

After correction for multiple comparisons, there were no differences in baseline characteristics between those that had data available at 12‐month and those that were enrolled in the program. Furthermore, binomial logistic regression was used to assess the predictors of members completing the Roczen program and no predictors were identified from the baseline characteristics.

## 4 DISCUSSION

This service evaluation of the Roczen program provides real‐world evidence of a novel, digitally enabled TRE intervention leading to clinically meaningful improvements in body anthropometry, eating behavior, and mental health in overweight and obese patients. The majority of members (89%) were public service workers who self‐referred via staff wellbeing and occupational health program to help improve their health. From this service evaluation, the Roczen program appears to have health benefits in this population,^34^ which could incur benefits to employers.

Interventions targeting the occupational health of a healthcare workforce are critical for potentially improving health, reducing absence rates and improving productivity. Digitally delivered occupational health interventions improve workers' health concerns and overall well‐being and may reduce metabolic risk.^17^, ^18^ Here, it is shown that the Roczen program leads to clinically significant and, importantly, sustainable weight loss at 12‐month in public sector workers. Mean percentage weight loss was 9.2%, with over 76% of members reducing their weight by ≥5%.^35^ Notably, at 12 months nearly half of the members lost ≥10% weight loss, while 15.9% achieved a BW loss of ≥15%. A 5% reduction is classed as clinically significant BW loss; however, further reductions have been shown to have greater improvements in health. For instance, 5%–10% improves mobility, while ≥15% weight loss has been linked to T2D remission and improvements in hepatic steatosis.^35^ Further evaluation is required to determine the direct of impact of the Roczen program on other important health‐related outcomes including glycemic control, T2D remission, lipid profile, and liver health, as improvements may partly be driven by weight‐independent mechanisms influenced by TRE.^20^, ^36^

The Roczen program appears comparable to other NHS tier 2 and 3 weight management program. Recent data from Tier 2 and Tier 3 weight management services showed mean aggregated weight loss of 2.2–12.4 kg, with 32%–51% of completers achieving ≥5% weight loss.^37^ Data from the digital stream of the NHS Diabetes Prevention Program revealed a mean weight reduction of 3.1 kg.^38^ Roczen is also comparable to other digitally delivered lifestyle interventions, where care is delivered by dietitians or health coaches via digital applications rather than clinicians.^39^, ^40^ Our retention rates was comparable to those in other Tier 3 specialist obesity program, with drop out ranging from 10% to 78% at the end of follow‐up.^41^ Overall, this service evaluation shows encouraging outcomes when compared to well established in‐person and digitally delivered NHS program.

Twelve‐month outcome data showed WC, an indicator of central adiposity, was reduced by >10 cm. Central adiposity is independently linked to an increased risk of T2D, non‐alcoholic fatty liver disease (NAFLD) and early death.^42^, ^43^ Although we did not define NAFLD at baseline or liver function within the service evaluation, there is a likelihood that the improvements in body anthropometry may be of benefit and incur potential improvements in glycemic control.^44^

In members living with T2D, glycemic control was significantly improved at 3 and 6 months, by approximately 10 mmol/mol equivalent to 1%. Despite this being in a relatively small subset of the membership, these show promising results regarding the impact of the Roczen program on glycemic control. Furthermore, members with T2D reduced weight by nearly 10% at 12 months (Table S2), which is clinically significant, not least because weight loss in those with T2D is usually inferior to those with prediabetes or normoglycaemia.^45^ Importantly, weight loss is a key predictor of T2D remission as demonstrated in the DIRECT and DIADEM‐1 trials.^46^, ^47^, ^48^ Although changes in glycemic control at 12 months were not formally reported due to challenges in obtaining data during COVID‐19, there is considerable potential for concomitant impact on glycemic control and BW in patients with T2D.^35^

Furthermore, of interest, females at 12‐month lose relatively greater amounts of BW compared with men. It is unclear as to the reason for this difference at this time—it may relate to greater data capture of women at 12‐month but may also reflect that TRE as a dietary method is an effective method for weight in females more than in men. Further evaluation is warranted.

Our data demonstrate that the Roczen program effectively reduces systolic and diastolic BP in the short term up to 9 months, but these effects were not maintained at 12‐month despite the reductions being similar. This likely reflects the smaller number of members and intra‐variability of BP readings at 12 months. Although the changes in BP were relatively small, ranging between approximately 3–5 mmHg, reductions of 5 mmHg in SBP have been suggested to reduce the risk of major cardiovascular events by 10%.^49^ At a population level, a 3 mmHg change in SBP has been suggested to prevent or postpone 11,000 coronary heart disease deaths over 7 years,^50^ showing that even relatively small reductions in BP can have substantial cardiometabolic benefits. Notably, previous research has highlighted that 3 months of TRE in people with the metabolic syndrome leads to improved cardiometabolic health parameters.^51^

The Roczen program applies multiple evidence‐based BCTs, including goal setting paired with self‐monitoring, feedback, motivational interviewing, and peer‐to‐peer social support.^39^ It can be hypothesized that these BCTs may underpin the improvements in mental health and weight loss maintenance in those completing 12‐month. Furthermore, remote but empathic social engagement, as afforded through the novel mentoring aspect of the Roczen program, likely drive improvements.^52^

People living with obesity and T2D have an increased risk of poorer mental health, including depression and anxiety.^53^, ^54^ Importantly, the members showed small though significant improvements in depression and anxiety symptoms at periods throughout the program. While program members tended toward low levels of depression and anxiety at baseline, there were a clinically significant number at baseline reporting either moderate to severe depression or anxiety, respectively. At 12‐month, no member appeared to report either severe or moderately severe depression, potentially showing improvement in those with the poorest mental health.

There remains concern that engaging in fasting and restrictive diets may increase the development of disordered eating, specifically BED.^55^ An important finding from this service evaluation was that binge eating scores did not deteriorate and in fact improved over the 12‐month program. This would show the idea that eating disorder risk is not impacted within behavioral weight management interventions offered alongside healthcare support, and that such clinically supervised program may in fact may be of benefit.^56^

Our service evaluation has several strengths. Firstly, a large proportion of the members were public service workers, with this representing a novel intervention in this population and offering a novel and deliverable intervention to improve workplace health for this population. Secondly, the Roczen program which is a medically led, digital TRE program featuring peer‐support mentoring, showed the feasibility of delivery and clinical efficacy of this approach. Finally, our data show the program's potential efficacy across a diverse population including a variety of ethnic groups.

Our service evaluation has limitations. Despite the large sample size that enrolled, there were challenges in obtaining self‐reported data at standardized time points from members, resulting in missing data. This meant that a pragmatic approach needed to be used to gather sufficient data; therefore, as described in the methodology plus and minus 2 weeks was used for outcome data and 12 weeks for HbA1c. However, this is not just a problem with our service evaluation, with over half of the participants in the NHSE digital DPP program missing weight data at 6 months^57^ Nevertheless, having adjusted for missing weight data, the weight loss remained clinical and statistically significant and appears comparable, if not greater, to other digital program offered by the NHS.^38^

## 5 CONCLUSIONS

This service evaluation provides evidence that a clinically led, digitally enabled TRE program, Roczen, can be efficacious within people living with overweight and obesity, the majority of whom work within the public sector. With workplace health being a governmental priority, this shows the potential use of this type of medical program within occupational health services to improve the health of employees. Further evaluation is warranted with a larger sample to determine the impact on glycemic control and other cardiometabolic parameters of interest, while also looking at the impact this has on workplace absences and quality of life.

## AUTHOR CONTRIBUTIONS

Barbara M. McGowan, Adrian Brown, Dipesh C. Patel, Jonathan T. C. Kwan, Siri Steinmo, Ling Chow conceived the study. Adrian Brown and Ling Chow reviewed the data. Adrian Brown was responsible for the data analysis with support from Barbara M. McGowan, Laurence J. Dobbie, Dipesh C. Patel, Jonathan T. C. Kwan, Siri Steinmo, and Ling Chow. Barbara M. McGowan takes responsibility for the oversight of the work. Adrian Brown, Laurence J. Dobbie and Laura Falvey wrote the initial draft manuscript. All authors contributed to data interpretation, and the writing of the manuscript. All authors contributed to critical revision of the manuscript and gave final approval.

## CONFLICT OF INTEREST STATEMENT

AB reports honoraria from Novo Nordisk, Office of Health Improvement and Disparity, Johnson and Johnson and Obesity UK outside the submitted work and is on the Medical Advisory Board and shareholder of Reset Health Clinics Ltd. DP reports personal fees from Astra Zeneca, Boehinger Ingelheim, Eli Lilly, MSD, Novo Nordisk, and Sanofi non‐financial support from Novo Nordisk outside the submitted work. He is a member of the Medical Advisory Board and a shareholder of Reset Health Clinics Ltd. DP is an executive committee member of the Association of British Clinical Diabetologists (ABCD) in the UK and member of the CaReMe UK group. BM is a member of the Medical Advisory Board and a shareholder in Reset Health and also performs Advisory and educational work for Novo Nordisk, Sanofi, Johnson and Johnson, Orexigen, Amgen and Advisory work for Novo Nordisk, Lilly, Pfizer, Boehringer Ingelheim and Johnson & Johnson. LJD does not declare any conflicts of interest. LF and LC are employees and shareholders of Reset Health Clinics Ltd.

## Supporting information

Supporting Information S1

## Data Availability

Data for the study are available upon reasonable request to the corresponding author.
