# Supplementary material for: Real‐world data of a digitally enabled, time‐restricted eating weight management program in public sector workers living with overweight and obesity in the United Kingdom: A service evaluation of the Roczen program
Source: Obes Sci Pract. 2024 Feb 9;10(1):e730. doi: 10.1002/osp4.730 (PMC10858326; doi:10.1002/osp4.730)
Supplement: Supplementary file 1 — Supporting Information S1 [file OSP4-10-e730-s001.docx]

**Supplementary Materials**

**Contents**

- **Page 2**; Supplementary Information 1: Inclusion / Exclusion Criteria for the Roczen Programme
- **Page 3**; Supplementary Table S2 Changes in Key Outcome at each time points over 12-months
- **Page 3**; Supplementary Table S3: Comparison of body weight Change by pre-diabetes and type 2 diabetes
- **Page 4**; Supplementary Table S4: Comparison of body weight Change in people living with or without obesity
- **Page 4**; Supplementary Table S5: Comparison of body weight Change by sex
- **Page 4**; Supplementary Table S6: Adjusted for missing data using last observation brought forward
- **Page 4**; Supplementary Table S7: PHQ-9 and GAD-7 Categories at each time point over 12-months

**Supplementary Information 1: Inclusion / Exclusion Criteria Defined for the Roczen Programme**

**Inclusion criteria**

- Over 18 years old
- Living with Overweight or obesity based on BMI cut-offs specific to ethnicity

**Exclusion Criteria**

- Safety Critical
  - Insulin
  - Sulphonylureas
  - Great than hypoglycaemic events
- Standard Criteria
  - Major adverse cardiovascular event in the last 6 months
  - Uncontrolled Heart Arrthymia
  - Currently uncontrolled Thyroid Disease
  - Cancer, or other malignancy, currently undergoing active treatment
  - Type 1 Diabetes Mellitus
  - Previously diagnosed with an eating disorder (bulimia nervosa, anorexia nervosa, binge eating disorder)
  - Liver Cirrhosis
  - Chronic Kidney Disease Stage IV or V
  - Uncontrolled Psychiatric Disorder
  - Suicidal Ideation
  - Previous Bariatric Surgery (not including those who have undergone gastric band / previously had gastric balloon removed)
  - Ileostomy
  - Body Mass Index > 55kg/m^2^
  - Active Inflammatory Bowel Disease (i.e. Crohn’s Disease or Ulcerative Colitis)
  - Women planning to conceive within the next 6 months
  - Glycated Haemoglobin >10%
  - Alcohol or drug dependency
  - Binge Eating Score of greater than 27 at onboarding

**Supplementary Table S1 Changes in Key Outcome at each time points over 12-months**

|  | ***n*** | **Baseline** | **3 months** | **6 months** | **9 months** | **12 Months** | ***p* Value** |
| --- | --- | --- | --- | --- | --- | --- | --- |
| Weight (kg) | 82 | 97.4 (19.0) | 89.7 (16.9) | 90.1 (19.1) | 88.9 (17.6) | 89.1 (20.0) | <0.001 |
| BMI (kg/m^2^) | 82 | 35.0 (5.7) | 32.4 (5.3) | 32.4 (5.5) | 31.9 (5.4) | 32.5 (6.5) | <0.001 |
| Waist circumference (cm) | 65 | 107.0 (14.0) | 98.8 (12.7) | 99.7 (13.0) | 97.9 (12.3) | 97.9 (14.0) | <0.001 |
| Systolic blood pressure (mm Hg) | 40 | 127.2 (13.5) | 122.7 (12.9) | 124.4 (12.7) | 124.4 (11.4) | 125.6 (13.1) | 0.061 |
| Diastolic blood pressure (mm Hg) | 40 | 79.6 (9.6) | 77.0 (10.1) | 77.0 (10.0) | 76.8 (9.0) | 78.5 (9.5) | 0.185 |
| PHQ9 | 28 | 4.0 (2.0, 8.0) | 2 (0, 4) | 2 (0, 4) | 3 (1, 6.5) | 3.5 (1.3, 5.8) | 0.372 |
| GAD7 | 28 | 3.0 (1.0, 7.0) | 1 (1, 7) | 2 (0, 5) | 2, (0, 5) | 3.5 (0.0, 5.8) | 0.695 |
| BES | 28 | 12.9 (8.0) | 6.9 (6.1) | 7.7 (7.4) | 9.2 (7.5) | 7.0 (6.6) | 0.006 |
| TFEQ Total | 27 | 45.0 (13.6) | 33.7 (14.2) | 33.8 (12.5) | 38.0 (12.1) | 34.8 (13.5) | 0.192 |
| TFEQ Restraint | 28 | 2.23 (0.6) | 2.45 (0.61) | 2.49 (0.54) | 2.52 (0.64) | 2.6 (0.5) | 0.006 |

Data are in n (%), mean (SD). Median (IQR) n, number; BMI, body mass index; kg/m^2^, kilograms per metre squared, kg, kilograms; PHQ9 = Patient Health Questionnaire. GAD7 = Generalised Anxiety Disorder Questionnaire. BES = Binge Eating Scale; mmol/L, millimoles per litre; mm Hg, millimetre of mercury, cm, centimetre, n, number

**Supplementary Table S2: Comparison of percentage body weight Change by pre-diabetes and type 2 diabetes**

|  |  | **Prediabetes** | | | **Type 2 Diabetes** | | |  | **All other members** | | |  | | |  |  |
| --- | --- | --- | --- | --- | --- | --- | --- | --- | --- | --- | --- | --- | --- | --- | --- | --- |
| **Time point** | ***N*** | ***n*** | **Weight Change (%)** | **95% CI** | ***n*** | **Weight Change (%)** | **95% CI** |  | ***n*** | **Weight Change (%)** | **95% CI** | |  | ***p* Value** | | |
| 3 months | 317 | 35 | -7.9 (4.2) | -9.3 to -6.4 | 33 | -9.1 (4.2) | -10.6 to -7.6 |  | 249 | -7.3 (4.4) | -7.9 to 6.8 | |  | 0.074 | | |
| 6 months | 204 | 29 | -8.2 (4.6) | -10.0 to -6.5 | 27 | -11.2 (6.0) | -13.7 to -8.9 |  | 148 | -8.8 (5.8) | -9.8 to -8.0 | |  | 0.094 | | |
| 9 months | 138 | 22 | -7.8 (4.6) | -9.9 to -5.8 | 16 | -11.1 7.0) | -14.8 to -7.3 |  | 100 | -10.0 (6.3) | -11.2 to -8.7 | |  | 0.226 | | |
| 12 months | 82 | 12 | -8.2 (4.4) | -11.1 to -5.5 | 10 | -9.8 (6.8) | -14.7 to -5.0 |  | 60 | -9.3 (7.2) | -11.2 to -7.5 | |  | 0.849 | | |

One-way ANOVA used to determine impact of pre-diabetes and type 2 diabetes on weight loss. Bonferroni adjustment used for post-hoc analysis. kg = kilogram, 95% CI = 95% confidence interval, mean (SD) = standard deviation,, p = p-value, n, number

**Supplementary Table S3: Comparison of percentage body weight Change in people living with or without obesity**

|  | **Living with obesity** | | | **Living without obesity** | | |  |
| --- | --- | --- | --- | --- | --- | --- | --- |
| **Time point** | ***n*** | **Weight Change (%)** | **95% CI** | ***n*** | **Weight Change (%)** | **95% CI** | ***p* Value** |
| 3 months | 262 | -7.8 (4.3) | -8.3 to -7.3 | 55 | -6.4 (4.4) | -7.6 to -5.3 | 0.035 |
| 6 months | 175 | -9.5 (5.7) | -10.3 to -8.6 | 29 | -6.8 (5.5) | -8.9 to -4.7 | 0.017 |
| 9 months | 119 | -10.1 (6.2) | -11.3 to -9.0 | 19 | -7.5 (5.1) | -10.0 to -5.1 | 0.087 |
| 12 months | 67 | -9.7 (7.0) | -11.4 to 7.8 | 15 | -7.3 (5.4) | -10.4 to -4.3 | 0.230 |

One-way ANOVA used to determine impact of obesity on weight loss. Bonferroni adjustment used for post-hoc analysis. %, percentage, 95% CI = 95% confidence interval, mean (SD) = standard deviation, *p* = p-value, n, number

**Supplementary Table S4: Comparison of percentage body weight Change by sex**

|  | **Female** | | | **Male** | | |  |
| --- | --- | --- | --- | --- | --- | --- | --- |
| **Time point** | ***n*** | **Weight Change (%)** | **95% CI** | ***n*** | **Weight Change (%)** | **95% CI** | ***p* Value** |
| 3 months | 246 | -7.4 (4.3) | -8.0 to -6.9 | 71 | -8.0 (4.4) | -9.0 to -7.0 | 0.342 |
| 6 months | 150 | -9.4 (5.6) | -10.2 to -8.5 | 54 | -8.4 (6.0) | -10.1 to -6.8 | 0.300 |
| 9 months | 100 | -10.2 (6.2) | -11.4 to -9.0 | 38 | -8.7 (6.0) | -10.6 to -6.7 | 0.194 |
| 12 months | 65 | -10.1 (6.9) | -11.8 to 8.4 | 15 | -5.8 (5.1) | -8.5 to -3.3 | 0.020 |

One-way ANOVA used to determine impact of sex on weight loss. Bonferroni adjustment used for post-hoc analysis. kg = kilogram, 95% CI = 95% confidence interval, mean (SD) = standard deviation, p = p-value, n, number

**Supplementary Table S5: Weight change adjusted for missing data using last observation brought forward**

| **Time point** | ***n*** | **Weight change (kg)** | **95% CI** | ***p* Value** |
| --- | --- | --- | --- | --- |
| 3 months | 317 | -4.0 (5.0) | -4.4 to -3.6 | <0.001 |
| 6 months | 204 | -4.9 (5.9) | -5.4 to -4.4 | <0.001 |
| 9 months | 138 | 5.0 (6.1) | -5.5 to -4.5 | <0.001 |
| 12 months | 82 | -5.0 (6.0) | -5.4 to -4.4 | <0.001 |

kg = kilogram, NA = not applicable, 95% CI = 95% confidence interval, mean (SD) = standard deviation, p = p-value

**Supplementary Table S6: Glycated haemoglobin changes in people living with type 2 diabetes over 3 and 6 months.**

| **Time point** | ***n*** | **HbA1c (mmol/mol)** | **95% CI** | ***p* Value** |
| --- | --- | --- | --- | --- |
| Baseline | 76 | 58.8 (6.2) | 56.7 to 60.9 |  |
| 3 months | 15 | 48.4 (2.4) | 44.6 to 52.5 | <0.001 |
| 6 months | 11 | 47.8 (5.7) | 43.4 to 52.6 | 0.012 |

Mmol/mol, millimoles per mole, 95% CI = 95% confidence interval, mean (SD) = standard deviation, p = p-value, n, number

**Supplementary Table S7: PHQ-9 and GAD-7 Categories at each time point over 12-months**

| **Categorical** | **Baseline** | **3 months** | **6 months** | **9 months** | **12 Months** | ***p* Value** |
| --- | --- | --- | --- | --- | --- | --- |
|  | n=550 | n=250 | n=111 | n=65 | n=28 |  |
| **PHQ-9** |  |  |  |  |  |  |
| None | 284 (51.6) | 198 (79.2) | 84 (75.7) | 40 (61.5) | 19 (67.9) | 0.021* |
| Mild | 172 (31.3) | 41 (16.4) | 19 (17.1) | 16 (24.6) | 7 (25.0) |  |
| Moderate | 66 (12.0) | 9 (3.6) | 6 (5.4) | 6 (9.2) | 2 (7.1) |  |
| Moderately Severe | 23 (4.2) | 1 (0.4) | 1 (0.9) | 2 (3.1) | 0 (0.0) |  |
| Severe | 5 (0.9) | 1 (0.4) | 1 (0.9) | 1 (1.5) | 0 (0.0) |  |
|  |  |  |  |  |  |  |
| **GAD-7** |  |  |  |  |  |  |
| None | 324 (58.9) | 199 (79.6) | 83 (74.8) | 45 (69.2) | 18 (64.3) | <0.001* |
| Mild | 168 (30.5) | 43 (17.2) | 20 (18.0) | 15 (23.1) | 8 (28.6) |  |
| Moderate | 37 (6.7) | 8 (3.2) | 7 (6.3) | 3 (4.6) | 2 (7.1) |  |
| Severe | 21 (3.8) | 0 (0) | 1 (0.9) | 2 (3.1) | 0 (0.0) |  |

PHQ-9, depression, GAD-7 anxiety score, n, number, *indicate difference over time between ordinal data
